# Epistasis: Just say no!

**DOI:** 10.1016/j.jbc.2026.113463

**Published:** 2026-08-19

**Authors:** Natalie M. Jaeger, Brennan A. Fitzgerald, Michael J. Harms

**Affiliations:** 1 Institute of Molecular Biology, University of Oregon, Eugene, Oregon, USA; 2 Department of Biology, University of Oregon, Eugene, Oregon, USA; 3 Department of Chemistry and Biochemistry, University of Oregon, Eugene, Oregon, USA

**Keywords:** epistasis, non-additive, mutant cycle, Genetics, model-fitting, high-throughput experiments, Biochemistry, biophysics, mechanistic model

## Abstract

Epistasis, where the effect of a mutation depends on other mutations, has been observed in virtually every quantitative biochemical feature examined. Despite its ubiquity, epistasis is often difficult to explain mechanistically. Its interpretation is thus often limited to phenomenological descriptions of synergy or antagonism. Here, we argue that epistasis quantifies the mismatch between experimental observations and a model for how mutations should sum together. Put differently: it is a fit residual. And like any fit residual, the presence of epistasis is a call to action, not an interesting observation in itself. It signals that the model is inadequate, not (necessarily) that the mutations physically interact. We propose that biochemists and biophysicists should pivot away from analyzing epistasis and into fitting mechanistic models that connect genotype to phenotype. Such models describe the effects of mutations as perturbations to underlying biochemical parameters—stability, binding affinity, catalytic rate—and combine those perturbations on mechanistically appropriate scales. Compared to a standard epistasis analysis, this approach reduces the number of free parameters, improves extrapolative power, and yields interpretable insights into structure-function relationships. We illustrate how this framework works in practice with studies covering a broad range of molecular systems and biochemically relevant scales. By reframing epistasis as a starting point rather than as a final observation, biochemists are uniquely positioned to build predictive models of mutational effects grounded in the physical principles governing biomolecular function.

Epistasis is when the effect of a mutation changes in different genetic backgrounds. It complicates protein design (1, 2), is a key factor shaping evolutionary trajectories (1, 3), and is mined as a source of information for understanding biological architecture (4).It has been reported in myriad biochemical features: enzyme activity (5, 6, 7, 8, 9, 10, 11, 12), binding reactions (12, 13, 14, 15, 16, 17), metabolic pathways (18), stability (19, 20, 21), condensate formation (22), and even chromophore color (23). The literature contains a veritable zoo of named forms of epistasis: threshold (24), environmental (9), diminishing-returns (11, 25), sign (25, 26), reciprocal-sign (27), permissive (28), ensemble (17, 29), and more.

How should biochemists and biophysicists make sense of this ubiquitous phenomenon? How can it be used to shed light on molecular mechanism?

We argue that epistasis is best viewed as a measure of model fit. It quantifies the mismatch between experimental observations and a model for how mutations should sum together. The model is often implicit: usually, an assumption that the effects of mutations should add. We argue that biochemists, however, are uniquely positioned to replace the implicit assumption of additivity with a mechanistic model for how mutations should combine. Viewed this way, epistasis is a starting point for further investigation, not an end point. The goal becomes eliminating epistasis through a better biochemical model, not characterizing epistasis as an end in itself.

In this, we are writing specifically to mechanistic biochemists and biophysicists. Other fields have their own field-specific perspectives. In evolutionary biology, for instance, epistasis in fitness can be a powerful framework for understanding the evolutionary process (4). But, for those interested in molecular mechanism, we believe the goal should be to eliminate epistasis.

In this review, we describe how one can view quantitative epistasis as a fit residual, and work through some of the consequences of that observation. We then flesh out how we think epistasis should be addressed mechanistically and highlight studies that use this framework. By recontextualizing epistasis with biochemical mechanisms, we ground it in fundamental principles of biomolecules instead of statistical correlations. This perspective improves our ability to both interpret and predict the effects of mutations.

## Quantitative epistasis

We’ll start by explicitly defining epistasis. There are several competing uses of the term, arising in part from historical differences in disciplines (4). For our purposes we will focus on quantitative epistasis—the form most often used by biochemists and biophysicists (2, 30). Imagine studying two mutations to a protein, a→A and b→B. We create a mutant cycle consisting of each mutation alone, as well as both mutations together (Fig. 1*A*). We then measure a quantitative feature *F* of each protein variant. *F* could be an energy, activity, rate, or wavelength maximum—any feature with a quantitative value. Epistasis is the difference in the effect of mutation a→A on *F* in either the “ab” or “aB” genetic backgrounds. Mathematically, this is:

(1) $$\varepsilon =\left(F_{AB}-F_{aB}\right)-\left(F_{Ab}-F_{ab}\right)$$

**Figure 1:**
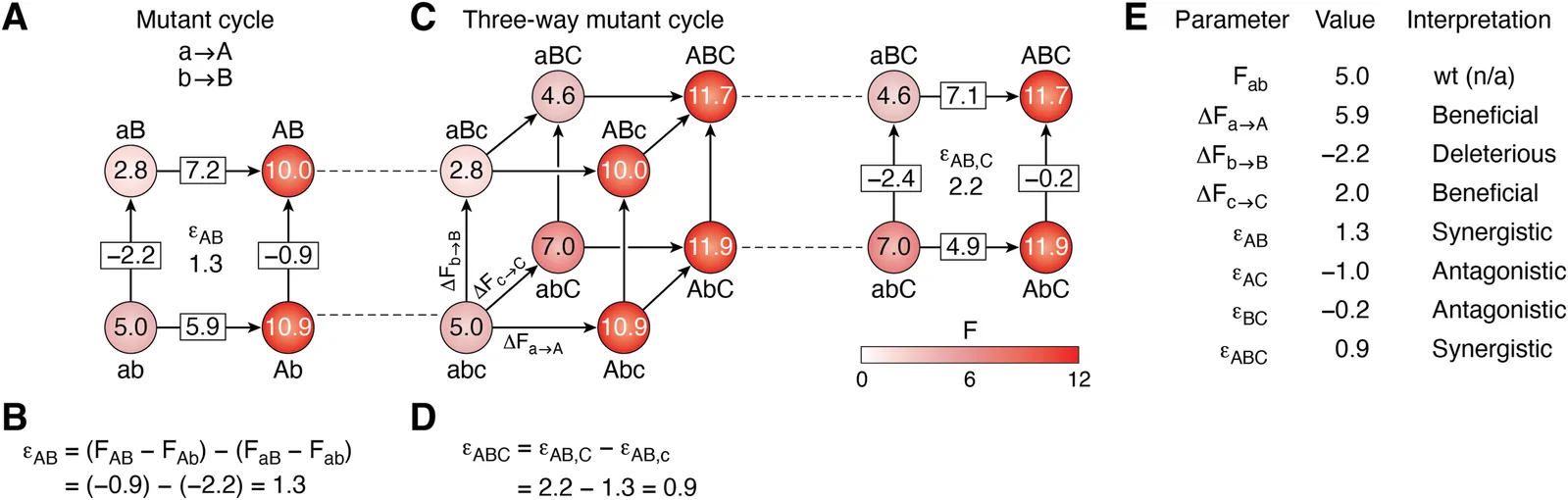
**Deconstructing mutations into epistatic effects.***A*, a mutant cycle between mutations a→A and b→B. Genotype is indicated next to each node. The number and color (*white*-to-*red*) on each node indicates the value of the quantitative feature *F*. *Arrows* indicate single mutations, with the quantitative effect of each mutation in the *white box* (*e.g.*, for ab→Ab: 10.9–5.0 = 5.9). The epistasis between the mutations is shown in the center of the cycle. *B*, the calculation used to extract ε_AB_ from the cycle shown in *panel A*. *C*, a three-way mutant. The front face is the same as *panel A*; the back face shows the same cycle in the c→C background. The *arrows* corresponding to the individual effects of each mutation in the abc background (*e.g.*, ΔF_a→A_) are indicated on the three-way cycle. *D*, the calculation used to extract ε_ABC_ from the three-way cycle in *panel C*. *E*, all eight epistatic parameter values extracted from the three-way cycle, with their interpretations.

where each F is the quantitative feature of genotype AB, aB, and so on We see quantitative epistasis if ε is not zero (after accounting for the uncertainty in our measurements). In Figure 1*B*, we see a synergistic interaction between the a→A and b→B. Quantitative epistasis has been observed in every quantitative biological feature at which someone has cared to look (1, 5, 6, 7, 8, 9, 10, 12, 13, 14, 15, 16, 17, 18, 20, 21, 28). But what does it mean?

## Epistasis as a physical interaction?

The simplest, and likely most common, interpretation of epistasis is as an “interaction.” This is somewhat tricky because “interaction” is used in different senses in different context (2, 30). In statistical or quantitative genetics, an interaction is often purely mathematical—the epistasis itself is the statistical “interaction”. But for someone considering molecules, an “interaction” is generally physical: the combination of two mutations results in epistasis because the amino acids touch one another, directly or indirectly. In what follows, we will exclusively consider physical interactions, viewed from the lens of biochemistry and biophysics.

Sometimes, interpreting epistasis as a physical interaction works well. Consider mutating two residues involved in a protein ion-pair or nucleic acid base-pair. One mutation changes the effect of another because of physical forces between the residues (2, 30, 31, 32, 33, 34). But epistasis is often much harder to interpret (1, 35). What is the nature of the physical interaction between epistatic mutations at distant sites (1, 17, 35, 36, 37, 38)? Or epistasis that changes with altered solution conditions (9, 18)? Or that occurs between two different enzymes in a pathway (11, 18)?

A physical interaction becomes even harder to rationalize if one considers epistasis between more than two mutations (5, 39, 40). If one measures a three-way mutant cycle (three mutations measured individually, in pairs, and then in combination; the cube in Fig. 1*C*) one can describe epistasis between three mutations. This manifests as a change in pairwise epistasis with the introduction of a third mutation. In Figure 1*C*, the epistasis between a→A and b→B changes from 1.3 to 2.2 with the introduction of mutation c→C (Fig. 1*D*). This difference (0.9) is three-way epistasis. Like pairwise epistasis, three-way epistasis is extraordinarily common (39, 40). Although it can sometimes be explained *via* a three-way cluster of contacts, the physical nature of a three-way interaction is often obscure.

Interpretation becomes even harder for more than three mutations. For example, O’Maille *et al.* built all 2^9^ combinations of nine mutations to a terpene synthase and then measured catalytic activity, finding many high-order interactions (41). In what way do nine mutations physically interact? This is not an isolated example. High-order epistasis is a common feature of genotype-phenotype maps (39, 40). Like pairwise epistasis, it appears whenever someone cares to look. Does every high-order epistatic term report on a physical interaction between the mutations?

## Epistasis as a polynomial

The fact that high-order epistasis is rampant suggests that epistasis is often reporting something besides direct physical interactions. But what? The key to answering this question is recognizing that standard epistatic models are polynomials. This has been discussed rigorously elsewhere (42, 43), so we will focus primarily on the implications of this fact.

One way to see the polynomial nature of these models is by counting the number of model terms and the number of genotypes. These numbers go up 1:1. A mutant cycle with four genotypes (ab, Ab, aB, and AB) requires four mathematical terms: the starting value of *F*, the individual effects of each mutation, and the epistasis between them (ε_AB_). A three-way mutant cycle has eight (2^3^) genotypes and eight epistatic terms: the starting value of *F*, three individual effects, three pairwise effects (ε_AB_, ε_AC_, ε_BC_) and one three-way effect (ε_ABC_) (Fig. 1*E*). This continues to all orders of epistasis. An N-way mutant cycle has 2^N^ genotypes and 2^N^ epistatic terms.

Another way to see the polynomial nature of these models is to note that a model with all terms captures all variation in the *F*. Put differently, a complete epistatic model with all terms will have an R^2^ of 1.0 when compared to the observations. It behaves like fitting a polynomial to any collection of points: add as many parameters as datapoints, and we can make the model go through every point.

Finally, the polynomial nature of this analysis is clear in the interpretation of the model terms. These are shown for our example mutant cycle in Figure 1*E*. Epistasis is “synergistic” if it has a positive sign; it is “antagonistic” if it has a negative sign. These are precise statements about the parameters themselves, but do not provide any physical or mechanistic meaning. This would be equivalent to noting the signs of the coefficients on a standard polynomial—not incorrect, but also not providing mechanistic information.

Fitting a polynomial model to genotype data has the same problems as fitting a polynomial to other sorts of data. Figure 2 shows three biochemical datasets: a ligand binding curve, a Michaelis–Menten plot for enzyme kinetics, and a protein folding/unfolding chevron. We can fit polynomials with the same number of terms as datapoints to each dataset (Fig. 2, red lines). Although the R^2^ of each fit is 1.0, the fit itself has limited utility: the model does not provide mechanistic insight, the values of the model parameters are not interpretable, and the model describes the experimental data but cannot be used to predict new data. (Note the wild oscillations between data points).

**Figure 2:**
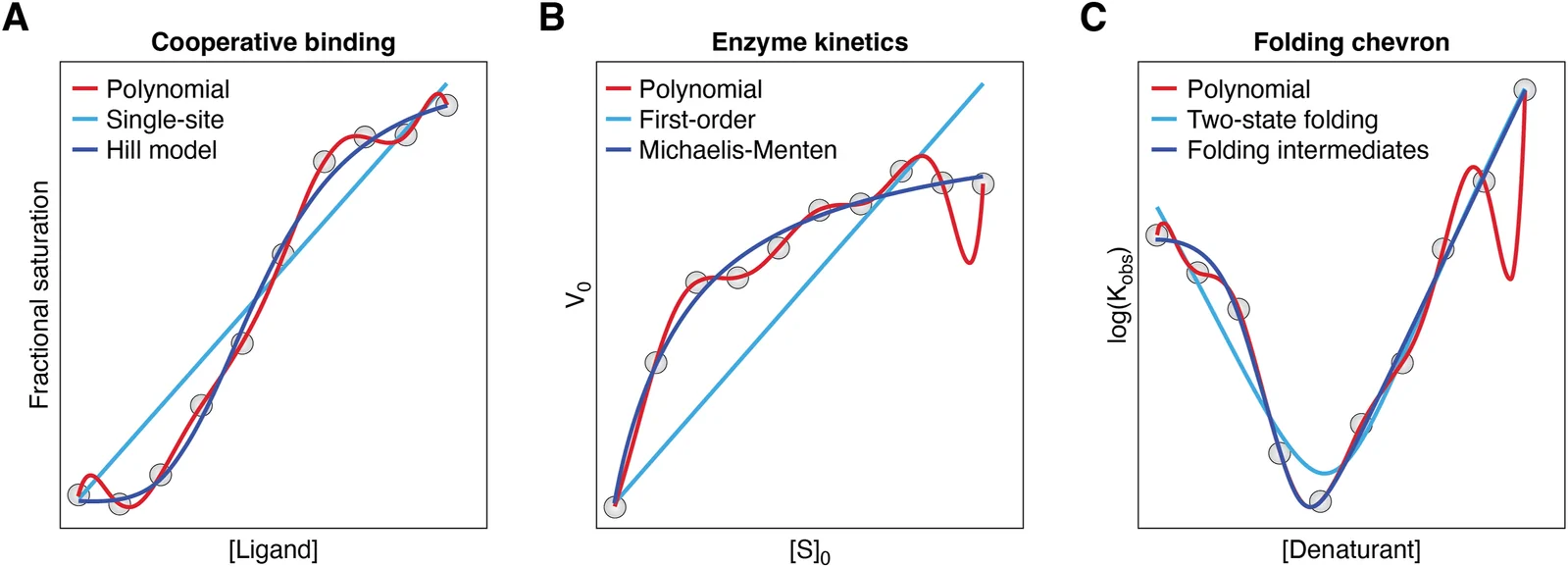
**Examples of mechanistic models for fitting biochemical datasets.** Each panel shows a classic biochemical dataset. *A*, binding shown as fractional saturation *versus* ligand concentration. *B*, enzyme kinetics shown as initial reaction velocity (V_0_) *versus* initial substrate concentration ([S]_0_). *C*, a protein folding chevron shown as log unfolding/folding rate *versus* denaturant concentration. The *red line* shows a ninth-order polynomial fit to each dataset. The *light blue* lines show a simple mechanistic model fit to each dataset (*left-to-right*: single-site binding, first-order kinetics in [S]_0_, and a two-state folder). The *dark blue* lines show a mechanistic model fit to each dataset that more accurately reflects the underlying mechanism than the simple model (*left to right*: a Hill model, a model that characterizes cooperative binding, with n = 4, a Michaelis–Menten curve, and a protein that folds through an intermediate). All data are simulated.

The primary utility of a polynomial model of epistasis is the same as a polynomial fit to other sorts of data: it can be used for interpolation. For example, if one has measured *F* for 50 of the 256 possible eight-way mutants to a protein, one might fit a low-order polynomial epistasis model to the limited data, then interpolate to estimate values of *F* for the remaining 206 genotypes. This has been done, with some success (37, 40, 44), but even the interpolative power of these models has proven mixed because identifying the appropriate epistatic order for the interpolation is challenging (38, 45).

But even if an epistasis model can be used for interpolation, it cannot be used for extrapolation. Training our model on eight mutations will tell us nothing about what happens when we add a ninth mutation. This is equivalent to fitting a polynomial to a biochemical dataset and trying to extrapolate outside the data range.

## Epistasis as a fit residual

Is there a more productive way to think about quantitative epistasis? To probe this, we can ask what epistasis really measures. As written in Equation 1, epistasis is a phenomenological score where we take a mathematical difference of differences to collapse four numbers into one. How can this be understood? We can start with some algebraic rearrangement. We can rewrite Equation 1 as:

(2) $$\varepsilon =F_{\text{AB}}-\left[F_{\text{ab}}+\left(F_{\text{Ab}}-F_{\text{ab}}\right)+\left(F_{\text{aB}}-F_{\text{ab}}\right)\right]$$

In this formulation, the right side of the equation in brackets is an additive prediction: “Take F_ab_ and add the effects of each individual mutant in that background.” Viewed this way, epistasis is the difference between our observation (F_AB_) and an additive prediction.

The most direct meaning, in our view, is accessed with one more rearrangement:

(3) $$F_{\text{AB}}=\left[F_{\text{ab}}+\left(F_{Ab}-F_{ab}\right)+\left(F_{aB}-F_{ab}\right)\right]+\varepsilon$$

Our epistasis equation has now become the equation that sits at the heart of model regression (37). In this expression, we have data (F_AB_), a model we think might describe the data (the terms in brackets), and our residuals (ε), which measure how well our model reproduces our data. Framed this way, quantitative epistasis is simply a fit residual—a measure of how well an additive model describes our observations. This is not new—this is how Fisher initially defined epistacy in 1918 (46)—but the implications of this interpretation are often lost.

What are the implications of viewing epistasis as a fit residual? Most importantly, a high fit residual is a call to action rather than an interesting observation on its own. The light blue lines in Figure 2 show simple mechanistic models fit to each of the biochemical datasets. In each case, a biochemist would note the high fit residual and infer that they needed a different model. (Imagine the alternative: a paper where the authors showed any of the light blue fits in Figure 2, noted the mismatch between the model and data, and then moved on without further comment!).

Further, a high fit residual would typically inspire fitting a new *mechanistic* model. For the examples in Figure 2, we replace a simple mechanistic model (light blue) with a more complicated mechanistic model (dark blue). We exchange single-site binding with cooperating binding sites (Fig. 2*A*), first-order kinetics with saturating enzyme kinetics (Fig. 2*B*), and two-state folding with a folding intermediate (Fig. 2*C*). These mechanistic models were derived from biochemical principles like linked equilibria and rate laws, meaning that the parameters are meaningful. Furthermore, if one mechanistic model fits the experimental data better than another, it gives evidence supporting the mechanism implied by that model.

A mechanistic approach contrasts strongly with a polynomial approach (Fig. 2, dark blue *versus* red lines). Biochemists generally avoid fitting polynomials to their data because such models provide no mechanistic insight. Epistatic models are no different. They are effectively polynomials in residuals. Is there residual variation after accounting for additive effects? Split the residual into pairwise residuals and call this pairwise epistasis. Does any residual remain? Split those values further into higher-ordered residuals. This allows one to fit an arbitrarily complicated collection of genotypic data, but the coefficients are uninterpretable.

Thus, a biochemist should treat epistasis no differently than any other fit residual. The presence of epistasis tells us that an additive model is inadequate to describe the data. Its existence is a call to action: an indicator that says to use a different model.

## A better approach

If epistasis is a residual, how should we analyze quantitative genotype/observable data with multiple mutations? Our approach can be summarized with five principles.

• Globally fit a single model to all genotypes and observations
• Connect genotype to observable with a mechanistic model that has a small number of physically meaningful parameters.
• Describe the effects of mutations as perturbations to these parameters, not as perturbations to the measured feature.
• Combine the effects of mutations on a mechanistically informed scale. Free energies should be added, equilibrium constants should be multiplied, and so on
• Do not include interaction terms just to improve the fit. Any added term should be mechanistically interpretable (or at least on path to being interpretable).

Figure 3 illustrates this approach for a hypothetical transcription factor driving expression of a reporter in response to an activating ligand. In this simple example, we observe the quantitative reporter output *F* at a single ligand concentration for eight combinatorial genotypes (Fig. 3*A*).

**Figure 3:**
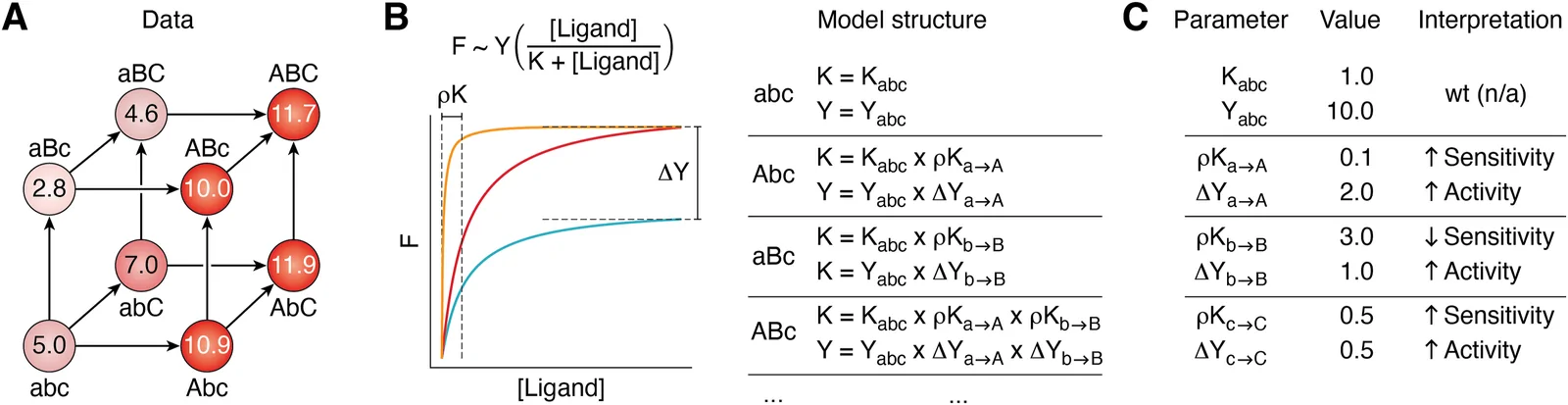
**Analyzing a transcription factor mutagenesis screen dataset using a mechanistic model.***A*, *Circles* show the value of the quantitative feature *F* for combinatorial genotypes between abc and ABC. Data are identical to those shown in Figure 1*C*. *B*, the equation indicates how *F* depends on maximum activation (Y) and sensitivity (K). The graph shows how changing Y or K alters *F*. The table shows how parameters combine in four of the eight genotypes in *panel A* (abc, Abc, aBc, and ABc). ΔY terms measure additive shifts in activation (*e.g.*, ΔY_a→A_ = (Y_Abc_ – Y_abc_)). ρK terms measure multiplicative shifts in sensitivity (*e.g.*, ρK_a→A_ = (K_Abc_/K_abc_)). *C*, parameter values that reproduce the data in *panel A*, with their mechanistic interpretations.

Like most biochemical experiments, we have an expectation for how mutations might alter the transcription factor: they can change its sensitivity to ligand (*K*) or they can change the maximum reporter output (*Y*) (Fig. 3*B*). We need eight terms to describe these genotypes: *Y* and *K* for the starting “abc” genotype, and then the effect of each point mutation on *Y* and *K*. For a genotype with multiple mutations, we combine the effects of mutations without adding any interactions (Fig. 3*B*). We anticipate that the effects of mutations to *Y* should sum, so we add their effects (*e.g.*, Y_ABc_ = Y_abc_ + ΔY_a→A_ + ΔY_b→B_). Equilibrium constants multiply, so we take the product of their effects (*e.g.* K_,ABc_ = K_,abc_ × ρK_a→A_ × ρK_b→B_, where ρK_a→A_ = K_Abc_/K_abc_). When the parameter values in Figure 3*C* are combined as shown in Figure 3*B*, they reproduce the experimental data. We can interpret the effects of the mutations mechanistically using the values of these terms. For example, mutation a→A increases sensitivity and activity, while mutation b→B decreases sensitivity while increasing activity (Fig. 3*C*).

We can contrast this to an epistatic analysis. The data in Figures 3*A* and 1*B* are the same. In Figure 1, we found that these data exhibit extensive epistasis, with both synergistic (ε_AB_ and ε_ABC_) and antagonistic (ε_AC_ and ε_BC_) effects (Fig. 1*E*). This analysis also revealed specific individual effects of mutations: a→A and c→C are favorable; b→B is unfavorable (Fig. 1*E*).

There are several things to note in comparing these two approaches. First, the mechanistic model gives interpretations that can be rationalized structurally and tested independently (Fig. 3*C*). For example, the model indicates b→B lowers affinity. We can check this. Is the mutation near the ligand binding pocket? Does the mutation alter binding when tested *in vitro*? By contrast, the epistatic interpretations are purely phenomenological (Fig. 1*E*). We learned that b→B is deleterious but gained no insight into why. Further, the epistasis analysis misses the fact that the “deleterious” mutation b→B increased maximum activity, despite lowering affinity.

Second, there is no way to map the epistasis to the underlying biochemistry. Although it is true that there is a synergistic three-way interaction between the mutations, this is not the product of a three-way contact. Rather, this arises from the additive effects of each mutation passed through the nonlinear activation model in Figure 3*B*. This creates an asymmetry: we can map from the biochemical effects of the mutations on *K* and *Y* to the three-way “interaction” in transcriptional output. But we cannot go the other way: given only the three-way “interaction”, we cannot learn its biochemical origin.

Third, and finally, there is a massive difference in parameter scaling. Both models have eight parameters for eight observations. A natural way to increase the number of observations would be to measure *F* for each genotype at different concentrations of ligand. If we measured the same mutant cycle at eight ligand concentrations, we would have 8 × 8 = 64 observations for eight mechanistic parameters. By contrast, the epistasis model is phenomenological: we would have to add eight more parameters for each ligand concentration, giving a total of 64 parameters for 64 observations.

The difference in scaling is even more stark if we consider adding a new mutation. If we added a new mutation d→D and measured its combinatorial effects (abcd, abcD, AbcD, and so on), we would now have 16 genotypes. The number of parameters in the mechanistic model would have increased by 2, to 10 total. In contrast, the epistatic model would have increased in lockstep with the number of genotypes: 16 genotypes = 16 parameters. For the mechanistic model, the number of parameters scales by 2(N + 1) where *N* is the number of sites mutated. For the epistasis model, the number of parameters scales by 2^N^. For a 10-site system, there would be 22 biochemical parameters and 1024 epistatic parameters. For 20 sites, there would be 44 and 1,048,576. One might use a shrinkage method like LASSO or horseshoe priors to ignore parameters whose values are ∼0, but this is itself an implicit admission that the model is poorly conceived.

## Direct contacts as a model choice

With this view of epistasis in mind, it might be helpful to return to the simple case of two sites in direct, physical contact in a protein. We measure the value of feature *F* for the WT, single mutants and double mutants at those sites. We then plug our measurements into Equation 2 and observe a large value for ε. We naturally interpret this as being due to the physical interaction between them. One might argue that in this case ε is not a fit residual, it is an interaction. Although this might be practically true, sticking to interpreting epistasis as a fit residual forces us to be explicit in our mechanistic model. We must modify our model for how mutations should add up to account for our structural model of interaction. In this case, we could add an explicit interaction term “U” to Equation 3:

(4) $$F_{\text{AB}}=\left[F_{\text{ab}}+\left(F_{Ab}-F_{ab}\right)+\left(F_{aB}-F_{ab}\right)+U\right]+\varepsilon$$

In this expression ε = 0 because the physical interaction “U” accounts for the nonadditivity of the two single mutants. Although we admit this is pedantic, we believe the distinction between epistasis (ε, the mismatch between our model and our observations) and the mechanistic model parameters (U, a physical interaction) is powerful and worth preserving.

## Epistasis as a starting point

Mechanistic models have better interpretability, integrate new experimental data without adding more parameters, and have many fewer parameters overall. But how should we apply mechanistic models in practice? Here, we present a selection of studies that move beyond treating epistasis as the end point of study. Instead, epistasis was the starting point, leading to mechanistic hypotheses and experiments to test them. This resulted in deeper mechanistic insights, explanations of evolutionary innovations and a better understanding of genotype-to-phenotype maps.

### Stability-mediated effects in a nucleoprotein

In one classic study, researchers observed epistasis between the mutations L259S and N334H in the influenza nucleoprotein (19). When introduced alone L259S is deleterious and N334H is neutral. Together, however, they increase fitness and promote viral escape. This leads to a specific evolutionary trajectory, with the neutral N334H mutation occurring first, followed by L259S (Fig. 4*A*). Contact epistasis is not a likely mechanism because the residues are distant in the structure (Fig. 4*A*). Instead, this arises from changes in protein stability. L259S decreases stability and N334H increases stability (Fig. 4*B*). The function-altering mutation L259S can only be tolerated if the protein is prestabilized by N334H. By evaluating a mechanistic hypothesis, these researchers identified the underlying parameter—protein stability—that determined the evolutionary path taken by influenza.

**Figure 4:**
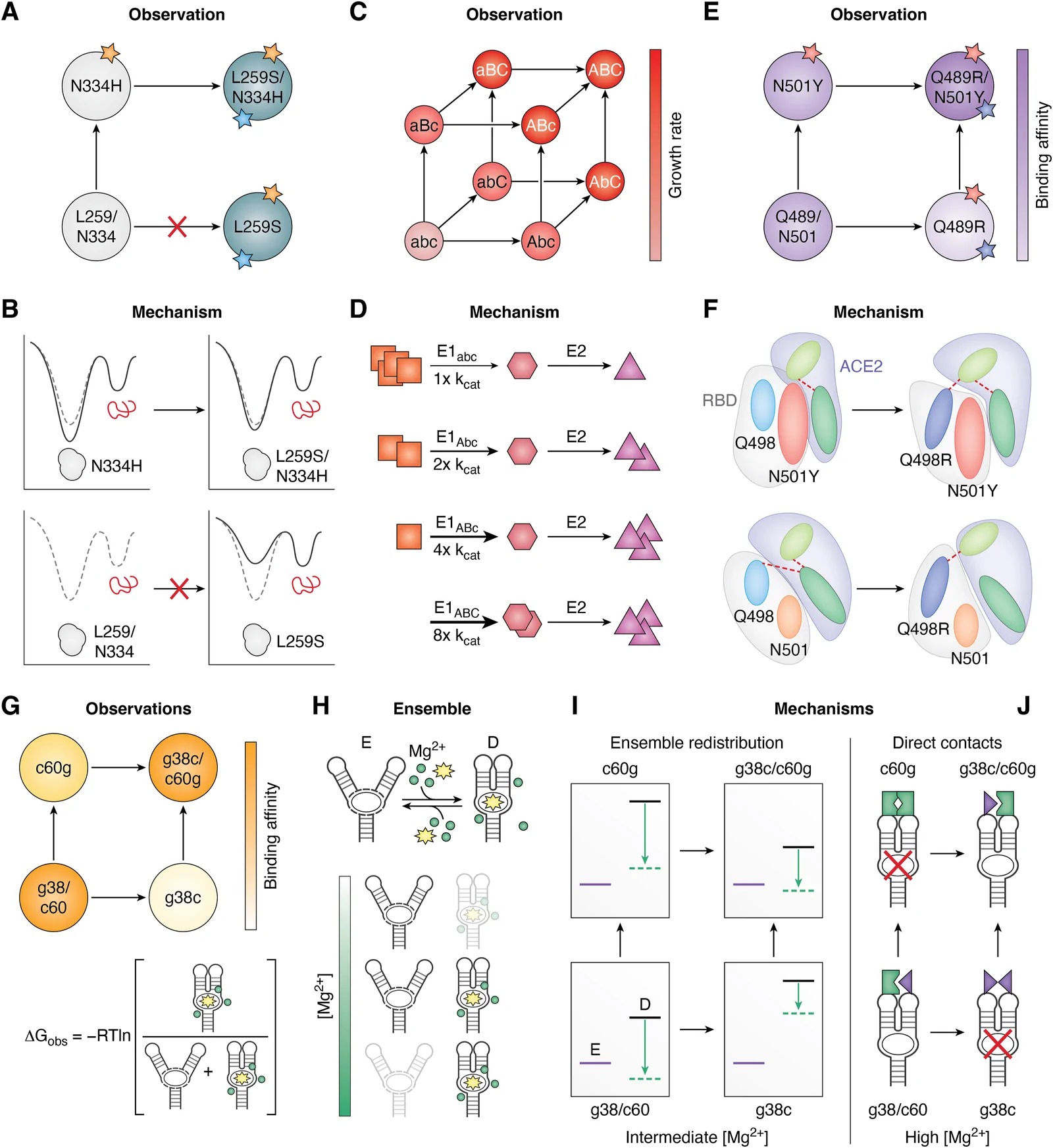
**Studying the combined effects of mutations in nucleoprotein stability, metabolic enzymes, receptor contacts, and contacts combined with ensembles in a riboswitch.** Different mechanistic studies of epistasis address stability, direct contacts, diminishing returns, and combinations of mechanisms. *A* and *B*, influenza nucleoprotein mutant cycle whose phenotype depends on stability mediated effects. *A*, *circles* are genotypes and each color indicates immune escape propensity: *low* (*gray*) or *high* (*teal*). Stars represent mutations. *Red* X represents the nonviable trajectory for this protein. *B*, changes in protein thermostability diagramed as energy landscapes between the folded structure and the unfolded. *C* and *D*, mutant cycle for SARS-CoV-2 receptor binding domain (RBD) which binds to ACE2 receptor in which direct contacts alter phenotype. *C*, RBD mutant cycle with increased affinity for ACE2 indicated by darker color. *Stars* represent mutations. *D*, shifts in polar contacts (*dotted red* lines) between RBD residues (*gray shaded*) and ACE2 residues (*blue* shaded) determine changes in binding. *E* and *F*, diminishing returns on *Escherichia coli* growth rate as the number of mutations to a metabolic pathway increases. *E*, a representative three-way mutant cycle of the accumulation of mutations whereas the observed growth rate plateaus. *F*, model of two enzymes E1 and E2 in this metabolic pathway where enzyme E1 freely evolves whereas enzyme E2 is biophysically constrained. The sizes of the arrows indicate enzymatic efficiency in k_cat_. As k_cat_ increases for E1, E2 becomes the rate limiting step. *G*-*J*, adenine riboswitch mutant cycle investigated for both direct contacts and ensemble effects. *G*, darker color indicates increase in binding affinity of the riboswitch to 2AP. *H*, ensemble of adenine riboswitch in two conformations *D* and *E*. The distribution of conformations in the ensemble changes as a function of magnesium (*green circle*). *I*, at low magnesium, epistasis arises from conformational redistribution because both *E* and *D* are populated. *J*, at high magnesium, epistasis arises from direct contacts because only D is populated. RBD, receptor binding domain.

Stability-mediated effects like these are a common cause of epistasis (47). Threshold epistasis (when mutations accumulate neutrally until the folded structure is sufficiently destabilized at some threshold) falls under this umbrella (19, 24, 48). It also includes ensemble redistributions in multiconformational proteins, and other effects on protein stability (17, 29). These stability-mediated effects are important to the fitness landscape of proteins. Mutations that increase stability allows for the accumulation of further mutations opening up new evolutionary trajectories (49, 50, 51). By identifying examples of stability-mediated mutational effects instead of assuming interactions we get stability principles for protein design, and insight into evolutionary processes.

### Diminishing returns in metabolic pathways

Many traits in biology are inherently nonlinear due to environmental or other constraints (51, 52, 53, 54). Even when the microscopic effects of mutations are additive, factors such as the energetic cost of transporting substrates can often impose nonlinearities that manifest as epistasis (54). One study used genome-scale modeling of metabolism to simulate the evolution of metabolic enzymes in *Escherichia coli* over evolutionary history (55). The researchers demonstrated that that the growth rate of *E. coli* consistently plateaued as mutations to a metabolic network accumulated (Fig. 4*E*): later mutations had a smaller effect on growth rate than earlier mutations. These researchers sought to mechanistically explain this observation. They hypothesized that enzymes are limited by other enzymes in the same metabolic pathway. Even if k_cat_ for E1 increased with every mutation, growth rate could be limited by a downstream enzyme (E2) (Fig. 4*F*). With this simple model, they recapitulated common observation of diminishing-returns epistasis in enzyme evolution with a mechanistic lens.

### Direct contacts: in a viral receptor binding domain

As one might expect, direct physical contacts between amino acids also cause epistasis (2, 17, 30, 33, 34). One example is found in the receptor binding domain (RBD) of SARS-CoV-2. Altering direct contacts facilitated the evolution of the SARS-CoV-2 RBD, which interacts with the host cell receptor ACE2 during infection. Looking at several strains, Starr *et al.* identified that the mutant pair Q498R/N501Y in the RBD substantially increased ACE2 binding affinity (56) (Fig. 4*C*). Alone, Q498R reduced binding to ACE2 whereas N501Y alone modestly increased it. Using known structures for the bound protein they found these mutations are part of a polar contact neighborhood that arises when ACE2 and RBD bind. Q498R and N501Y each differently reorient amino acids in this network. This disrupts the pattern of polar contacts between ACE2 residues and RBD residues (Fig. 4*D*). Introduced together these two mutations reposition other nearby residues to establish a new satisfactory local network of contacts. The new combination of salt bridges and polar contacts satisfies each member of the neighborhood. By taking a mechanistic approach, they were able to identify a specific insight into a network of residues critical to this virus’s evolutionary trajectory.

### Co-occurring mechanisms in a riboswitch

A mechanistic lens can also be used to take apart systems with multiple, interacting epistatic mechanisms (17, 31). For example, our lab investigated the interplay of direct contacts and conformational redistribution causing epistasis in the adenine riboswitch (17). This molecule fluctuates between the H and D conformations. Both magnesium and adenine preferentially bind the D conformation (Fig. 4*H*). Mutations g38c and c60 g have reciprocally epistatic effects on adenine affinity. Alone, both mutations are deleterious; together, they increase affinity (Fig. 4*G*).

In this system, one source of epistasis is a direct base-pair between positions 38 and 60 in the D conformation. The second source of epistasis is a redistribution of the conformational ensemble induced by these mutations. Ensemble redistribution causes epistasis when mutations with different effects on different conformations are introduced together. Directly analogous to the ensemble model of allostery, two mutations indirectly “interact” with one another by altering populations of different conformations of a macromolecule (57).

In the g38c/c60g mutant pair, ensemble redistribution contributed to epistasis at low magnesium concentration. Under these conditions, the D and E conformations have similar populations, exposing the separate effects of each mutation each conformation (Fig. 4*I*). Meanwhile, at high magnesium, when the population is fully in the D conformation, direct contacts were the primary cause of the epistasis (Fig. 4*J*). These kinds of experiments provide generalizable principles for how to identify when different mechanisms of mutational effects contribute the most to observed epistasis.

### Agnostic mechanistic models

The underlying mechanism behind mutational effects is not always known up front. This does not change the framework for modeling; however, Otwinowski *et al.* demonstrated the power of nonlinear models of mutational effects with unknown, but additive mechanisms (37). In the specific case of GB1, they examined binding data for mutations to 55 sites of the protein. Using a model that explicitly included a term for an unobserved additive trait inside a nonlinear function, they were able to explain most of the binding data. This kind of mechanistically-flexible or agnostic model is useful analysis on path to mechanistic understanding. It generates a starting point for generating hypotheses about the underlying mechanism(s) and enables predictive capacity for the effects of new combined mutations. Furthermore, these authors expand on their work to show that the remaining epistasis, or fit residual, could then be explained by specific unique interactions between mutation sites. This demonstrates the importance of moving away from studying epistasis as a phenomenon and moving toward a global model of mutation effects on an underlying mechanism. Even in the absence of mechanistic intuition, there is still more achieved by this framework than a simple epistasis model. One can also write out models that are partially agnostic. Work by Birgy. *et al.* demonstrated this in a deep mutation scan (DMS) of β-lactamase TEM-1. Their additive agnostic functional model with an explicit consideration of protein stability improved the prediction of combined mutant effects (47). This predictive power derived from mechanistic models, agnostic or not, highlights the utility of eschewing epistasis in understanding the effect of mutations.

### Interpretable machine learning models

Applying a mechanistic framework to the analysis of DMS data is an ongoing area of development. One exciting innovation in this field is MoCHI which was designed to overcome the problem of uninterpretable parameters using both the agnostic and mechanistic approaches described above (58). MoCHI takes DMS data and allows users to fit a model of unknown but additive mutant effects or a user-specified mechanistic model. The advantage is that this unknown additive model follows the finding that global epistasis is not interactions. Instead, it is a nonlinear relationship between phenotype and some additive mechanism of mutant effects. The additive terms capture the individual effects of mutations; the nonlinear phenotype map leads to the observed epistasis between them. Furthermore, this tool allows users to test different mechanistic models on large mutant library data. By grounding interpretation of mutations in global mechanisms, MoCHI applies the framework we have outlined here. This kind of tool improves the application of machine learning to DMS by providing interpretable global parameters.

## Reframing how we think about epistasis

As our ability to collect abundant data on the effect of mutations has improved, so has the importance of interpreting epistasis. We believe that the best way forward is to discard the idea of epistasis as a “something” and instead reframe it as a fit residual. This view is as old as epistasis itself (46). There is limited utility in defining epistasis as an interaction. It fails to generate predictive models and hinders mechanistic understanding. Focusing on epistasis means losing sight of the mechanisms that lead to nonadditivity.

Instead of focusing on epistasis, researchers should model the underlying mechanistic relationship between genotype and phenotype. The presence of “epistasis” points to the need for better mechanistic models. A mechanistic model limits the number of fit parameters and allows for interpretation of their values. Such models have been applied successfully to explain epistasis and gain detailed insight into the effects of mutations. This positions biochemists and biophysicists to make huge contributions, as they bring strong fundamental principles to the studies of the effects of mutations on sequence-structure-function relationships. These insights should be applied to understand the features that introduce nonlinearities in these relationships, centering on the physical effects of mutations. The field should “just say no” to epistasis and “just say yes” to models that capture the mechanisms that cause it.

## Conflict of interest

The authors declare that they have no conflicts of interest with the contents of this article. Please refer to cited articles for any data described in this article.
